# Supplementary material for: Diagnostic Potential of microRNAs in Extracellular Vesicles Derived from Bronchoalveolar Lavage Fluid for Pneumonia—A Preliminary Report
Source: Cells. 2022 Sep 22;11(19):2961. doi: 10.3390/cells11192961 (PMC9564323; doi:10.3390/cells11192961)
Supplement: Supplementary file 1 [file cells-11-02961-s001.zip › cells-1783753-supplementary.pdf]

**Supplementary Information**

Table S1 Summary of candidate miRNAs searched by articles

| Numeber | Search methods | No. of papers |
|---|---|---|
| **#1** | (((("pneumonia"[MeSH Terms]) OR ("Pulmonary Inflammation"[Title/Abstract])) OR ("Pulmonary infection"[Title/Abstract])) OR ("Lung Inflammation"[Title/Abstract])) OR (pneumonia[Title/Abstract]) | 291,850 |
| **#2** | (((MicroRNAs[MeSH Terms]) OR (microRNA*[Title/Abstract])) OR (miRNA*[Title/Abstract])) OR (miR*[Title/Abstract]) | 150205 |
| **#3** | #1 AND #2 = “miRNAs cluster 1” | 561 |

Table S2 Quantitative Real Time PCR Primers’ sequences

| Gene | Forward (5’-3’) | Reverse (5’-3’) |
|---|---|---|
| hsa-miR-17-5p | CAAAGTGCTTACAGTGCAG | |
| hsa-miR-193a-5p | GTCTTTGCGGGCGAGATGA | |
| hsa-miR-542-3p | TGTGACAGATTGATAACTGAAA | |

| | | |
|---|---|---|
| hsa-miR-16-5p | TAGCAGCACGTAAATATTGG | |
| hsa-miR-20a-3p | ACTGCATTATGAGCACTTA | |
| hsa-miR-27a-5p | CTTAGCTGCTTGTGAGCA | |
| hsa-miR-92a-3p | TATTGCACTTGTCCCGGCC | |
| hsa-miR-342-3p | TCTCACACAGAAATCGCAC | mRQ 3' Primer |
| hsa-miR-422a | ACTGGACTTAGGGTCAGAA | (Takara, Kyoto, Japan) |
| hsa-miR-423-5p | TGAGGGGCAGAGAGCGAGA | |
| hsa-miR-582-3p | TAACTGGTTGAACAACTGAA | |
| hsa-miR-885-5p | ATTACACTACCCTGCCTCT | |
| hsa-miR-193b-5p | GGTTTTGAGGGCGAGATGA | |
| hsa-miR-432-5p | TCTTGGAGTAGGTCATTGG | |
| hsa-miR-493-3p | TGAAGGTCTACTGTGTGCC | |
| hsa-miR-452-5p | AACTGTTTGCAGAGGAAAC | |
| hsa-miR-200b-3p | TAATACTGCCTGGTAATGATGA | |
| hsa-miR-34a-3p | CAATCAGCAAGTATACTGCC | |
| U6 | Takara, Kyoto, Japan | Takara, Kyoto, Japan |

**Supplementary Figures**

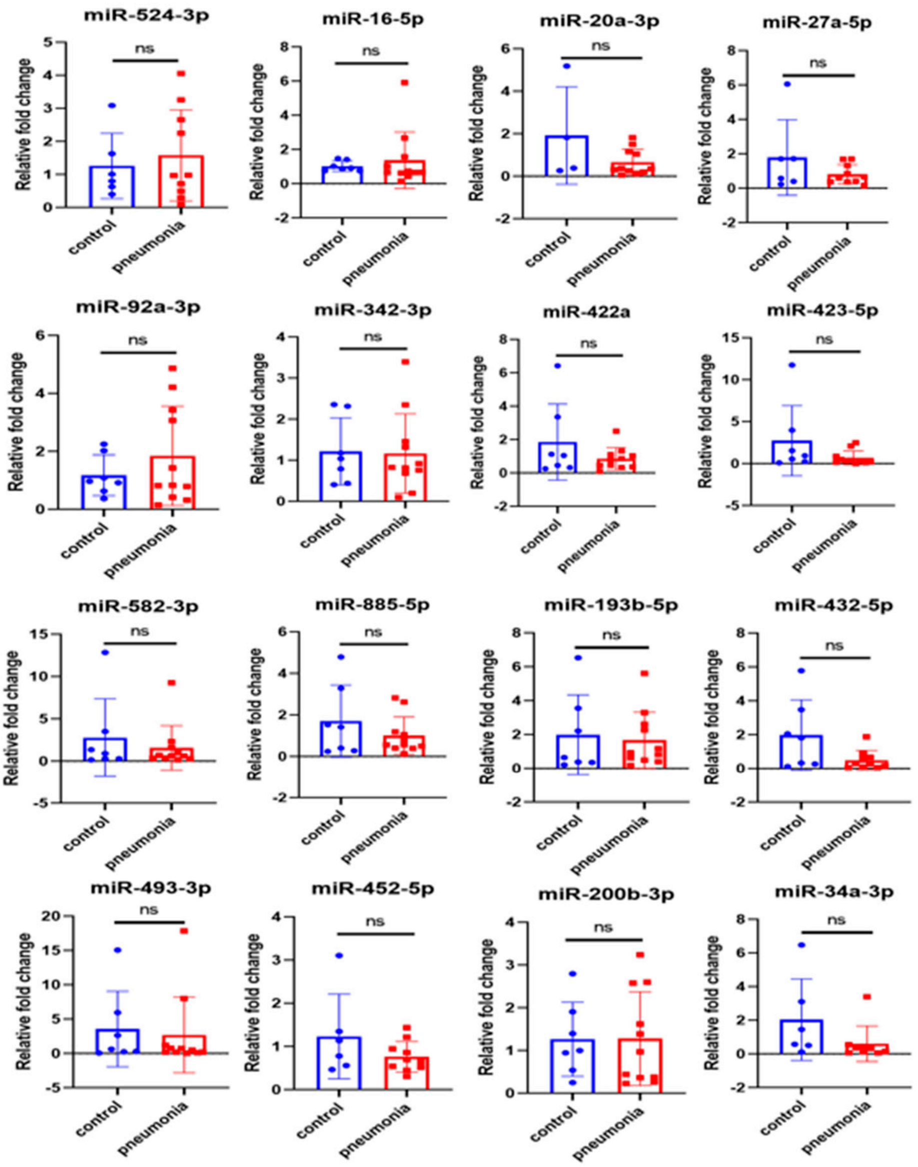


**Supplementary Figure S1.** Sixteen miRNAs in extracellular vesicles derived from bronchoalveolar lavage fluid (BALF−EVs) showed no statistical difference between pneumonia group(n = 12)and control group(n = 6). Data presented as a relative fold change for each miRNA. Box plots are displayed where the horizontal bar represents the median, the box represents the IQR, and the whiskers represent the maximum and minimum values. Comparisons made by Mann–Whitney U test. miRNA microRNA, IQR interquartile range

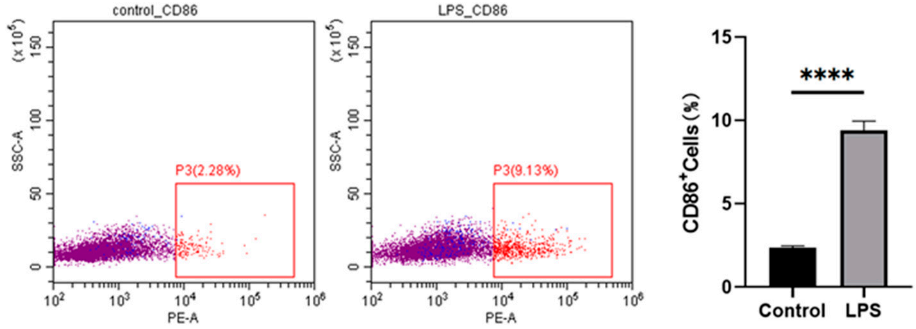


**Supplementary Figure S2.** The THP-1 derived macrophages (tMACs) were differentiated into M1 macrophages by stimulating them with 1 µg/mL of LPS for 24 h.(A) The percentage of M1 (CD86+) macrophages was estimated by flow cytometry (n = 3).
